# Design and Simulation of an Integrated Centrifugal Microfluidic Device for CTCs Separation and Cell Lysis

**DOI:** 10.3390/mi11070699

**Published:** 2020-07-20

**Authors:** Rohollah Nasiri, Amir Shamloo, Javad Akbari, Peyton Tebon, Mehmet R. Dokmeci, Samad Ahadian

**Affiliations:** 1 Department of Mechanical Engineering, Sharif University of Technology, Tehran 11365-11155, Iran; Rhnasiri90@gmail.com (R.N.); Akbari@sharif.edu (J.A.); 2 Center for Minimally Invasive Therapeutics (C-MIT), University of California—Los Angeles, Los Angeles, CA 90095, USA; peytontebon@g.ucla.edu (P.T.); dokmeci1@gmail.com (M.R.D.); 3 Department of Bioengineering, University of California—Los Angeles, Los Angeles, CA 90095, USA; 4 Department of Radiological Sciences, David Geffen School of Medicine, University of California-Los Angeles, Los Angeles, CA 90095, USA

**Keywords:** microfluidics, circulating tumor cells, cell separation, micromixer, cell lysis

## Abstract

Separation of circulating tumor cells (CTCs) from blood samples and subsequent DNA extraction from these cells play a crucial role in cancer research and drug discovery. Microfluidics is a versatile technology that has been applied to create niche solutions to biomedical applications, such as cell separation and mixing, droplet generation, bioprinting, and organs on a chip. Centrifugal microfluidic biochips created on compact disks show great potential in processing biological samples for point of care diagnostics. This study investigates the design and numerical simulation of an integrated microfluidic device, including a cell separation unit for isolating CTCs from a blood sample and a micromixer unit for cell lysis on a rotating disk platform. For this purpose, an inertial microfluidic device was designed for the separation of target cells by using contraction–expansion microchannel arrays. Additionally, a micromixer was incorporated to mix separated target cells with the cell lysis chemical reagent to dissolve their membranes to facilitate further assays. Our numerical simulation approach was validated for both cell separation and micromixer units and corroborates existing experimental results. In the first compartment of the proposed device (cell separation unit), several simulations were performed at different angular velocities from 500 rpm to 3000 rpm to find the optimum angular velocity for maximum separation efficiency. By using the proposed inertial separation approach, CTCs, were successfully separated from white blood cells (WBCs) with high efficiency (~90%) at an angular velocity of 2000 rpm. Furthermore, a serpentine channel with rectangular obstacles was designed to achieve a highly efficient micromixer unit with high mixing quality (~98%) for isolated CTCs lysis at 2000 rpm.

## 1. Introduction

Cancer currently stands as the second highest cause of deaths worldwide [1]. The prognosis of cancer patients significantly improves with early diagnosis. It is well established that most cancer patients die not from the primary tumor, but from metastatic tumors generated elsewhere. The origin of the metastatic tumors arises from cells that extravasate from the primary tumor into the blood stream. These cells, called circulating tumor cells (CTCs), that are shed from primary tumors into the bloodstream, can be isolated from blood samples to provide new methods for cancer diagnosis [2]. The separation of CTCs from the blood stream has grown as a research field due to the importance of understanding and monitoring the type of the metastatic stage of cancer [3,4]. It has been shown that CTCs in blood samples are a reliable indicator of cancer progression or drug response to cancer and the early detection of CTCs can enable early action [5].

Commercial cell separation techniques, including Magnetic-Activated Cell Sorting (MACS) and Fluorescent-Activated Cell Sorting (FACS), are powerful technologies that utilize the cell’s specific properties for identification and cell capture by tagging cells with specific antibodies and fluorescent dyes. However, these techniques suffer from some critical shortcomings, including high cost of equipment, need for expert users, and the requirement to label features with specific antibody [6]. Microfluidics is a proven technology that has been employed to create niche solutions to biomedical applications, such as cell separation and mixing, 3D bioprinting [7,8,9,10], and organs-on-a-chip systems [11,12]. The application of microfluidic technologies can address some of the limitations of mentioned commercial cell separation methods by using different physical mechanisms, including filtration- [13], hydrodynamic- [14], inertial- [15], deterministic lateral displacement (DLD)- [16,17], pinched flow fractionation (PFF)- [18], centrifugation- [19], dielectrophoresis (DEP)- [20], magnetic- [21], acoustic- [22], and optical-based approaches [23]. These methods can separate target cells from a heterogeneous cell population by exploiting the differences in the properties of the cells, including their size, density, shape, deformability, and compressibility, as well as their electric, magnetic, and optical properties. Microfluidic cell separation devices benefit from portability, low cost, small size, and blood compatibility [24]. In particular, label-free cell separation techniques decrease cell damage and eliminate the costly steps of cell labeling [25].

The operation of centrifugal microfluidic platforms depends on the manipulation of biological samples in microchannels on a rotating compact disk (CD), called lab on a CD (LOCD), at high speeds [26]. It exploits centrifugal and Coriolis forces as driving forces for fluid flow. Microchannels and reservoirs are integrated on a compact disk, and the platform rotates at a particular angular velocity. Different microfluidic functions, such as mixing, valving, and cell separation, can be incorporated on this platform and make the LOCD a promising technology for diagnostics and point-of-care applications [27]. Centrifugal microfluidic platforms have advantages over other microfluidic systems, including minimal required instrumentation using a rotor to pump liquids without direct contact with external hardware [28], elimination of syringe pumps, and the efficient removal of any disturbing bubbles or residual volume. Moreover, they benefit from inherently available density-based sample transportation and separation [29]. Centrifugal microfluidics have been utilized in numerous studies, in which the separation of blood components are needed. This includes plasma separation from blood cells [30] and the extraction of leukocytes from blood samples [31] and, additionally, to separate immune cells [19] and isolate CTCs from whole blood [32].

Inertial focusing is a label-free method and particles or cells are aligned and separated based on their size and density. This method employs inertial lift and drag forces in microchannels with different geometries including spiral [3], contraction–expansion [33,34], straight [35], and serpentine channels [36,37] to separate cells. Several groups have utilized inertial focusing to isolate CTCs from blood by using spiral [3,4], contraction–expansion [34], and centrifugal platforms [32,38,39]. Among the common inertial microfluidic cell separation devices, the spiral shape designs have received the most attention for target cell separation. For instance, Warkiani et al. [3] used a high-throughput spiral microfluidic device to separate CTCs from WBCs. Their proposed device works based on the inherent Dean vortex flows and inertial lift forces. In another publication, the group also showed that a trapezoidal cross-section channel creates higher Dean vortex cores in the outer wall. The device was used to trap smaller particles with higher efficiency and separation throughput, compared to rectangular cross-section channels [4]. In recent advances for spiral devices for capturing CTCs in an automated manner and high throughput, Zhang et al. [40] utilized a spiral microfluidic device to isolate MCF-7 cancer cells from human blood. One of the major concerns with the use of inertial focusing is the significant dilution of the sample required, which leads to large fluid volumes and longer processing times. Additionally, concentrating the samples after sorting leads to target cell loss and reduced sensitivity. Khoo et al. [41] developed an inertial method that simultaneously sorts and concentrates target cells by recirculating the target cell output into the sample source. Over time, excess volume is deposited into a waste reservoir, while the target cells are concentrated in a smaller fluid volume.

For cell separation using contraction–expansion channels, Lee et al. [33] exploited a microfluidic device composed of microchannel arrays for the label-free separation of cancer cells from whole blood. They reported the separation of cancer cells (MCF7) from whole blood with a recovery rate of 99.1% and a throughput of 1.1 × 10^8^ cells/min. Che et al. [34] proposed a high-throughput vortex chip with contraction–expansion channel arrays to separate CTCs by employing several parallelized channels and reservoirs to increase the population of rare CTCs with rapid flow rate (0.8 mL/min), high efficiency (83%), and high purity. For serpentine inertial device applications, Zhang et al. [37] presented a device to separate blood cells from plasma. Straight channels were also used in some methods of CTC separation [42,43]. More examples for inertial methods and other size-based separation methods of CTCs are reviewed by Hao et al. [44].

After designing a proper separation unit, a mixer unit is required to mix the separated target cells with lysis buffer to break down the cell membranes and extract DNA content for further study [45]. Mixing is one of the essential tasks in biological applications, and microfluidic systems can provide efficient micromixers for the mixing of a small amount of samples [26]. Microfluidic mixer platforms are usually categorized into two groups—passive and active. Passive mixing systems often work at low Reynolds numbers and their efficiency is based on the geometry of microchannels designed to increase the contact area of the fluids to maximize diffusion. These micromixers should be appropriately designed with sufficient length to achieve the desired mixing performance [27]. Among these systems, Wang et al. [46] examined the mixing process in a T–shaped tree-like microchannel and studied this system by both numerical and experimental methods and concluded that in low Reynolds number flows, the mixing efficiency was improved by increasing the number of T branches. Moheb et al. [47] examined mixing quality numerically and experimentally in T-shaped and cross-shaped microchannels. They concluded that cross-shaped structures with low volumetric flow rates had higher efficiency than the T-shaped structure.

Mixing is one of the essential steps in cell lysis [48]. Fluid carrying cells and fluid carrying lysis reagents should be uniformly mixed until the desired result is achieved. Microfluidic mixers can perform cell lysis in an integrated manner with a microfluidic cell separation unit. A common analytical method following the separation of CTCs is genomic and transcriptomic profiling. In order to extract the genetic content, the cell membranes need to be broken by mixing with lysis buffers [49]. This process provides access to intracellular substances, including DNA, proteins, and other related components for further study [50]. To this end, the design of an efficient micromixer is desired to reach a well-mixed mixture of separated CTC with the lysis buffer. Details of the design of the micromixer unit for cell lysis are discussed in the following sections.

The simulation and modeling of microfluidic devices is a useful method for predicting device performance and finding optimal properties and flow rates prior to fabrication and trial and error experimentation. Additionally, it improves the understanding of the mechanisms behind microfluidic flow and phenomena while providing insight into flow and particle/cell behavior inside microfluidic devices [25]. In this work, the numerical simulation of a centrifugal microfluidic platform for CTCs separation from blood in a size-based and label-free manner was carried out by using an inertial focusing approach on a CD. Since CTCs have greater size compared to other blood cells, an inertial cell separation method can be implemented due to its efficient and label-free characteristics. In the next stage, a micromixer was designed for cell lysis to extract the DNA content of these cells for further study. In the following sections, the details of each unit are investigated separately.

## 2. Materials and Methods

### 2.1. Description of the Proposed Platform Geometry and Function of Each Part

**Figure 1:**
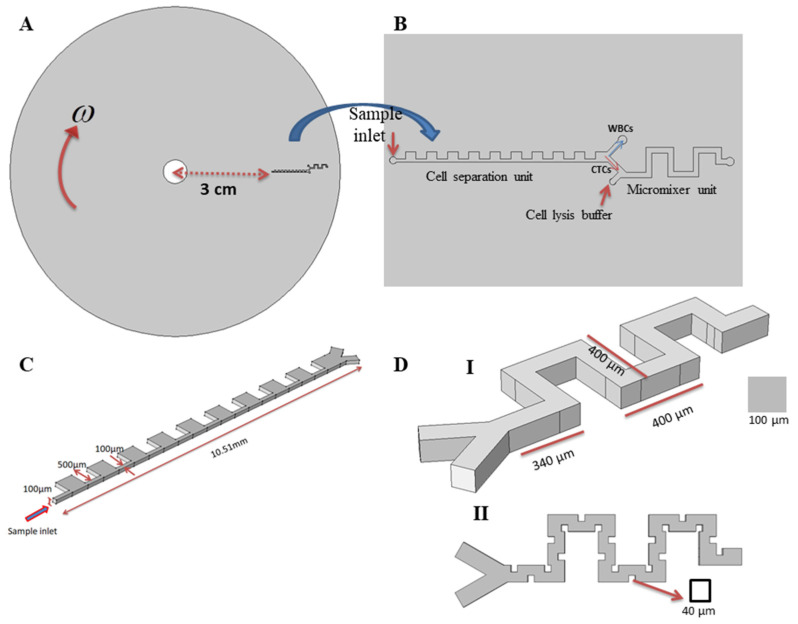
Proposed LOCD device. (**A**) Two-dimensional schematic of the designed centrifugal-based microfluidic biochip composed of serially arranged separator and mixer subunits. (**B**) Magnified view of the proposed device. (**C**) Cell separation unit, the CTCs are separated from the WBC cells by the implementation of inertial contraction–expansion arrays. (**D**) The mixer unit for cell lysis: (I) serpentine channel without obstacles and (II) with obstacles.

A two-dimensional (2D) schematic of our proposed chip is presented in Figure 1A. As can be observed, the platform is composed of two subunits in a serial arrangement in a clockwise rotating disk. Figure 1B provides a 2D top view of the subunits with more detail. Figure 1C presents the portion of the device designed to separate CTCs. In this segment, there is an inlet through which the blood sample containing CTCs is introduced into the channel. The separation unit is located 3 cm from the center radially. The sample enters the inlet channel and moves through a contraction–expansion channel, leading to two outlets. The lower outlet is designated for CTCs departure and the upper outlet is designed for the collection of WBCs. Subsequent mixing in the lower channel is attained by using a serpentine path, without any external forces to achieve mixing. The isolated CTCs suspension enters from one inlet and the lysis buffer is introduced into the micromixer unit from the second inlet. Figure 1D shows a schematic of the micromixer unit. As the two streams flow inside the mixing unit, two counter-rotating vortices are formed inside the channel to further mix the two flows. Both units are independent of any external force fields and are driven by fluid dynamic forces.

In order to study the proposed integrated microfluidic device with numerical simulation, governing equations of each part are explained in this section.

### 2.2. Governing Equations of Fluid Flow

Governing equations for fluid flow are Navier–Stokes equations (Equation (1)) for momentum conservation and the continuity equation for mass conservation (Equation (2)):

(1) $$\rho_{{}_{f}}\left(\partial_{t}+\vec{u}.\nabla \right)\vec{u}=-\nabla P+\mu_{f}\nabla^{2}\vec{u}+\vec{f},$$

(2) $$\nabla .\vec{u}=0,$$

in which $\vec{u}$ is the fluid velocity vector, *P* is the pressure, $\rho_{f}$ is the density of the fluid, $\mu_{f}$ is the dynamic viscosity, and $\vec{f}$ is the sum of all body forces, which in this study for a rotating platform on a CD includes the centrifugal force and Coriolis force.

In this study, the fluid is assumed to be Newtonian and incompressible. In general, in microfluidic devices, the flow regime is laminar, and the Reynolds number (Equation (3)) that expresses the ratio of inertial force to viscous force in laminar flow inside channels is less than 2300 (*Re* < 2300).

(3) $$Re_{C}=\frac{\rho_{f}U_{m}D_{h}}{\mu_{f}},$$

In calculating Reynolds number, *U_m_* is the average fluid flow velocity and *D_h_* is the characteristic length or hydraulic diameter of the channel that can be determined by equation (4), in which *H* and *W* are height and width of the channel, respectively.

(4) $$D_{h}=\frac{2W\times H}{W+H},$$

In a rotating platform, the centrifugal and Coriolis forces are considered in the $\vec{f}$ term in the above equation and can be summarized as in the following Equation (5) that can have three components in x, y and z directions:

(5) $$\vec{f}={\vec{f}}_{Centrifugal}+{\vec{f}}_{Coriolis}=-\rho_{f}\vec{\omega }\times \left(\vec{\omega }\times \vec{r}\right)-2\rho_{f}\vec{\omega }\times \vec{u},$$

in which $\vec{\omega }$ is an angular velocity vector and $\vec{r}$ is a radial location vector. Our simulation model is based on the clockwise rotation of the platform.

### 2.3. Governing Equations for Particle Tracking

In order to track particle (cell) paths through the proposed separation unit, Newton’s law of motion (Equation (6)) is solved to determine the motion of the particles.

(6) $$\frac{d(m_{p}v_{p})}{dt}=F_{total}=F_{Lift}+F_{Drag}+F_{Centrifugal}+F_{Coriolis},$$

In Equation (5), $m_{p}$ is the particle mass, $v_{p}$ is the velocity of particles. Lift, drag, centrifugal, and Coriolis forces are applied to cells/particles. The following equations describe the net centrifugal and Coriolis forces applied on cells/particles in a reference frame:

(7) $$F_{centrifugal}=-(\rho_{p}-\rho_{f})\frac{\pi d_{p}^{3}}{6}\vec{\omega }\times \left(\vec{\omega }\times \vec{r}\right),$$

(8) $$F_{Coriolis}=-(\rho_{p}-\rho_{f})\frac{\pi d_{p}^{3}}{3}\vec{\omega }\times {\vec{v}}_{p},$$

in which $\rho_{p}$ is the particle density. The following equation calculates the drag force based on Stokes’ drag law:

(9) $$F_{Drag}=3\pi \mu d_{p}v_{t},$$

in which $v_{t}$ is the relative velocity of the fluid to the particle [51].

In our proposed inertial microfluidic device, composed of contraction–expansion arrays, cells in fluid flow within the channels feel an inertial lift force due to a balance between shear-gradient and wall-induced lift forces (Equation (10)), and the resultant force is related to particle size (d_p_), Reynolds number ($Re_{C}$), and lateral positions in the channel cross-section (z) [52,53]. Moreover, the creation of secondary flow (Dean flow) as two counter-rotating vortices within contraction sections of this channel leads to exerting a drag force on the particles (*F_D_*), called Dean drag force that is presented in Equation (11). $U_{Dean}$ in this equation is the strength (velocity) of secondary flow. The balance between *F_L_* and *F_D_* forces in the mentioned channels determines the equilibrium positions of the cells for cell sorting [15].

(10) $$F_{L}=\frac{\rho_{f}U_{m}^{2}a^{4}{}_{\;}}{D_{h}^{2}}f_{L}\left(Re_{C},z\right),$$

(11) $$F_{D}=3\pi \mu U_{Dean}d_{p},$$

In the above equations, d_p_ is the diameter of a particle, and $f_{L}$ expresses the inertial lift force coefficient, which is related to the channel Reynolds number and the particle position along the channel cross-section (*z*).

A sudden change of the cross-sectional area of the channel perturbs fluid streams and creates an effect similar to Dean flow in a curved channel of constant cross-section. The curvature of the fluid streams induce Dean drag forces on the particles. Additionally, the particles are affected by inertial lift forces throughout the contraction areas as well as centrifugal and Coriolis force. These forces act on particles throughout the contraction–expansion arrays of the microchannel, and their force balancing specifies their equilibrium positions in the cross-section of the outlet channel [54].

In order to apply lift forces on particles, Ho et al. [55] proposed a formulation for inertial lift forces by investigating the motion of a sphere in a two-dimensional Poiseuille flow, and obtained an explicit formula for the lift force as an Equation (12), However, the model is restricted to a domain in which *Re* << 1, thus limiting its application to practical scenarios. Direct numerical simulation (DNS) is one of the most accurate methods for obtaining lift forces. Liu et al. [56] modified the formulation proposed by Ho et al. by using DNS and proposed a generalized formula for the inertial lift on a sphere as a function of the local flow field. This generalization is also adaptable to complex channel geometries as well as a wide range of Reynolds numbers [56].

The proposed formula by Liu et al. for wall-induced and shear gradient effect lift forces is Equation (13). In this equation *C*_1_ and *C*_2_ are correction coefficients that are obtained by using DNS simulation [56] that can be used for wide range of Reynolds numbers and aspect ratios (AR = W/H) of the channel, as shown in Table 1.

(12) $$F_{L}=F_{w}+F_{s}=\frac{\rho_{f}U_{\max}^{2}d_{p}^{4}}{H^{2}}\left(\beta^{2}G_{1}\left(s\right)+\beta \gamma G_{2}\left(s\right)\right)$$

(13) $$F_{L}=F_{w}+F_{s}=\frac{\rho_{f}U_{\max}^{2}d_{p}^{4}}{H^{2}}\left(C_{1}\beta^{2}G_{1}\left(s\right)+C_{2}\beta \gamma G_{2}\left(s\right)\right)$$

(14) $$C_{L}=C_{1}\beta^{2}G_{1}\left(s\right)+C_{2}\beta \gamma G_{2}\left(s\right)$$

**Figure 2:**
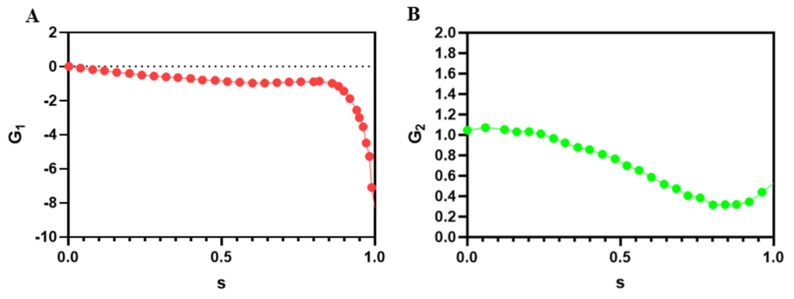
The functions (**A**) *G*_1_ and (**B**) *G*_2_ from [56].

The abovementioned formula (Equation (13)) involves the parameters of the local flow field *β* and γ that represents the dimensionless shear rate and the dimensionless shear gradient, respectively, as well as the global parameters $\rho_{f}$, *U*_max_, *H*, and *d_p_*. *H* is the distance between walls, *U*_max_ is the maximum channel velocity, and *G*_1_ and *G*_2_ are functions of nondimensional wall distance s. These functions are plotted in Figure 2. *s* is the nondimensionalized distance from the particle to the reference wall; the actual distance divided by H, so that 0 < *s* < 1 for particles in channel. The *β* and γ are normalized by *U*_max_ and *H*. The main characteristics of the formula remain unchanged unless Re is much higher than 100, which is rare in inertial microfluidics so it can be used for a wide range of Reynolds numbers [56].

The centrifugal and Coriolis forces have important roles in the LOCD device as they are the driving forces for fluid flow and other fluid dynamic forces originate from their existence. Here, we compared these forces with inertial lift, another important force in this study, according to the following Equations (15) and (16). With respect to our proposed application, the density of the cells (1050 kg/m^3^) is higher than the density of the carrier fluid (1000 kg/m^3^) and in the first terms in Equations (15) and (16) in the first parentheses, the magnitude of $\left(\rho_{p}-\rho_{f}\right)H$ and $\rho_{f}d_{p}$ are in the same order of magnitude because, although $\rho_{f}>\rho_{p}-\rho_{f}$ in the other side, the height of the channel is several times greater than particle diameters $\left(d_{p}<H\right)$. Therefore, the numerator and denominator in this parenthesis are in same order of magnitude. Moreover, the velocity of a rotating object at radial distance r is equal to rω and, since the fluid velocity originates from the rotation of the platform, it can be estimated that $U_{\max}∼v_{p}∼r\omega$. The following equations show that, for instance, at an angular velocity of 1000 rpm (~100 rad/s) and higher than this value, which is common for LOCD devices, each term in the numerator and denominator for second parentheses in Equations (15) and (16) are almost in the same order of magnitude. The same comparison can work for the third parenthesis. In summary, it can be concluded that, in our proposed application, the effect of centrifugal and Coriolis forces in particle motion has notable importance as does inertial lift force.

(15) $$\frac{F_{Centrifugal}}{F_{L}}\propto \frac{(\rho_{p}-\rho_{f})d_{p}^{3}r\omega^{2}}{\frac{\rho_{f}U_{\max}^{2}d_{p}^{4}}{H^{2}}}\propto \frac{(\rho_{p}-\rho_{f})H^{2}r\omega^{2}}{\rho_{f}U_{\max}^{2}d_{p}}\propto \left(\frac{(\rho_{p}-\rho_{f})H}{\rho_{f}d_{p}}\right)\left(\frac{H\omega }{U_{\max}}\right)\left(\frac{r\omega }{U_{\max}}\right)$$

(16) $$\frac{F_{Coriolis}}{F_{L}}\propto \frac{(\rho_{p}-\rho_{f})d_{p}^{3}v_{p}\omega }{\frac{\rho_{f}U_{\max}^{2}d_{p}^{4}}{H^{2}}}\propto \frac{(\rho_{p}-\rho_{f})H^{2}v_{p}\omega }{\rho_{f}U_{\max}^{2}d_{p}}\propto \left(\frac{(\rho_{p}-\rho_{f})H}{\rho_{f}d_{p}}\right)\left(\frac{H\omega }{U_{\max}}\right)\left(\frac{v_{p}}{U_{\max}}\right)$$

### 2.4. Governing Equations for Mixing

To study the mixing phenomena in the micromixer section, first the governing equations for fluid flow, including the Navier–Stokes and continuity equations, are solved. To determine the mixing behavior in the proposed micromixer, the concentration equation, called the convection–diffusion Equation (17) is solved to yield a concentration distribution for two reagents in the mixing unit.

(17) $$D\nabla^{2}c=\vec{u}.\nabla c,$$

In Equation (17), D is the diffusion coefficient of the species and c is the concentration.

To determine the mixing unit efficiency, we defined a parameter called mixing quality (*M.Q.*). To evaluate this parameter, first the mass average of concentration in specified cross-sections of the channel is calculated, and the deviation of concentration from the average value is attained. Then, this value is divided by the average value to normalize it. *M.Q*. is determined by subtracting the calculated value from one. Equations (18) and (19) were proposed to determine the mixing efficiency [57,58]:

(18) $$CoV=\frac{\sqrt{\frac{\sum {\left(c_{i}-c_{avg}\right)}^{2}}{N-1}}}{c_{avg}},$$

(19) M.Q.=1−CoV,

in which $c_{i}$ is the concentration of the sample, N is the number of points in which concentration is taken in calculations and $c_{\mathrm{avg}}$ is the average concentration. The mixing index M.Q. is used to quantify mixing performance, which varies between 0 (no mixing) and 1 (perfect mixing).

### 2.5. Numerical Method

All mentioned governing equations were solved with a lab-made, 3D unsteady code. It uses a Finite Element Method (FEM) solver that utilizes the Affine Invariant Adaptive Newton Codes. Pseudo time-stepping leads to stabilization and convergence through controlling the CFL (Courant–Friedrichs–Lewy) number. The aforementioned code was employed to solve the Navier–Stokes equations for fluid flow and the convection–diffusion equation for the concentration distribution. The Coriolis and centrifugal forces were applied as body forces, which were added as a source term to the Navier–Stokes equations. These body forces drive the fluid motion. The boundary conditions for the inlets and outlets of the proposed cell separation platforms and mixer units are considered to be atmospheric pressure. For the mixer unit, defined concentrations on the inlet boundary conditions were applied. The Galerkin method was utilized to approximate the nonlinear governing equations with a system of ordinary differential equations. The weak formulations of the convection–diffusion and Navier–Stokes equations were obtained by the discretization of the weak forms in a finite dimensional space. The Eulerian method was used for the 3D transient domain for tracking particles [59].

Unstructured meshes were used to discretize the computational domain of separation and mixer geometries. For the fluid flow, the Navier–Stokes and continuity equations were numerically solved for laminar flow in the discretized domain. For particle tracing, the transient equations of motion were solved with a 0.00005 s time step. For the modeling of particle motion, the dominant forces, including drag force, wall induced lift force, shear gradient lift force—proposed by Liu et al. [56] as mentioned in the above section—and Coriolis and centrifugal forces, are applied to the equation of motion for particles. For the mixing section, the boundary conditions were set to specified concentrations at inlets and, after solving the Navier–Stokes equations by the abovementioned method, the convection–diffusion equation for the concentration distribution of the reagents inside the micromixer unit were solved in a steady-state condition.

To simulate the separation of CTCs from blood cells, two sizes of particles with diameters of 20 µm and 10 µm, corresponding to the approximate average diameter for CTCs and WBCs [43], respectively, were used. The density of both cells was established as 1050 kg/m^3^. Centrifugal and Coriolis forces were considered as body forces due to the rotation of the system, and inertial lift forces were applied as dominant forces for lateral migration of particles. The proposed geometry for the separation unit is shown in Figure 1C. In this unit, it was assumed that the flow is 3D, and particle–particle interactions were neglected due to the dilution of blood samples in the inertial method in practical applications. The simulations were performed for different angular velocities ranging from 500 rpm to 3000 rpm. The particles contained by the fluid (blood sample) entered from the inlet and passed through the contraction–expansion array. Based on the lift, drag, centrifugal, and Coriolis forces applied, the particles were sorted, based on their size, into different outlets. By using the appropriate boundary conditions and generating a proper mesh for the proposed separation geometry, the simulations were performed for different angular velocity.

In order to study the mixing process, a serpentine channel, as shown in Figure 1D, was used to form secondary flows through the channel. Obstacles are arranged inside the channel to increase disturbance to achieve high mixing efficiency for cell lysis processes. Simulations were performed for a serpentine micromixer with and without obstacles inside the channel, and we found that adding obstacles increases the regions in which the secondary flows form—also because of obstacles— two fluids have more contact length with each other that enhance the diffusion of two fluids within each other and leads to a significant improvement in the mixing quality. After generating an unstructured mesh for a mixing unit with a maximum and minimum size of 3.1 μm and 1.7 μm, respectively, and determining the mixing quality at the outlet of the mixer unit, mesh independency was verified. In the simulations, 675,032 grids were used for simulation.

The centrifugal and Coriolis forces were applied as source terms to the Navier–Stokes equations to apply rotating effects to the proposed mixer model. The distance between the CD center and the mixing section was designed to be 4.3 cm from the center of rotation. Square-shaped obstacles with dimensions of 40μm×40μm were used inside the channel. By applying proper boundary conditions for mixer units as 0 mole/m^3^ for inlet 1, the entrance of the fluids (plasma) with isolated CTCs, and 1 mole/m^3^ for inlet 2 for the entrance of the lysis buffer, the concentration equation was solved. For the walls of the microchannel, the no-slip condition was used for the fluid flow study and properties of water, including 1000 kg/m^3^ for density, 0.001 Pa.s for viscosity, were used. The no-mass flux boundary condition was considered for all walls of the microchannel and diffusivity of $1.67\times 10^{-9}m^{2}/s$ was considered for mass transport studies.

## 3. Results and Discussion

**Figure 3:**
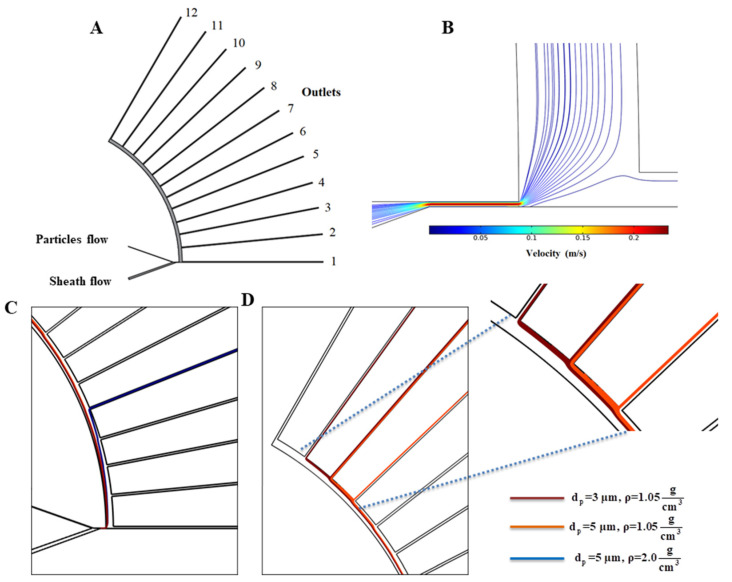
(**A**) An illustration of the Morijiri et al. [38] model, (**B**) Numerical simulation results for the velocity field in narrow region. (**C**,**D**) particles’ paths for different particles at different sections of the platform.

In order to validate our particle tracing simulation, our proposed numerical approach was used to simulate the experimental work on a centrifugal microfluidic particle separator by Morijiri et al. [38]. As shown in Figure 3A, this separator consists of two inlets and twelve outlets. Particles enter from the upper inlet and sheath flow enters from the lower inlet. We simulated the fluid flow and particle tracing for their proposed platform. Figure 3B shows the velocity field with streamlines in the narrow region. Three particles were used in their experiment including: polystyrene particles with a diameter of 3 µm and density 1.05 g/cm^3^; polystyrene particles with diameters of 5 µm and density of 1.05 g/cm^3^; silica particles with a diameter of 5 µm and density of 2.0 g/cm^3^. After passing through the narrow region of the channel, these particles were sorted by applied centrifugal force as well as wall-induced lift forces and followed distinct streamlines to specified downstream outlets. The authors reported that, at an angular velocity of 750 rpm, 98% of silica particles left the channel through outlet number 5 and about 87% of polystyrene particles with a diameter of 3 µm left the channel from outlets 9–11. In their experiments, about 80% of polystyrene particles with diameters of 5 µm were collected in outlets 9 and 10. Our simulations for particle trajectories are shown in Figure 3C and Figure 3D and our results agree with their experimental data, predicting that larger particles go through outlet 5 and particles with a diameter of 3 µm and 5 µm with lower densities leave the channel through outlets 9, 10, and 11.

**Figure 4:**
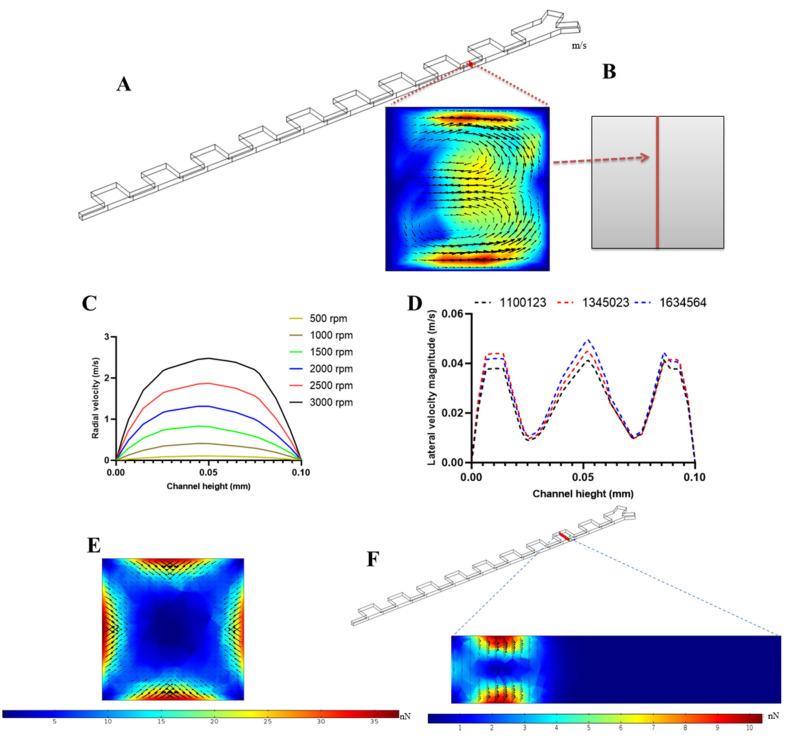
Simulation of Flow field for the proposed cell separation unit (**A**) formation of two counter-rotating vortices in a cross-section in the contraction region. (**B**) Defined vertical line in the cross-section. (**C**) Radial velocity profile for the different angular velocity of disk. (**D**) Mesh independency analysis for cell separation unit at three different mesh densities for lateral fluid velocity magnitude along the vertical red line in the middle of the channel cross-section. (**E**) Lift force magnitude distribution and its vectors in a cross-section of contraction section for 20 µm particles. (**F**) Lift force magnitude distribution and its vectors in a cross-section of expansion section for 20 µm particles.

As illustrated in the above sections, our proposed cell separation chip worked based on the specific geometry of the channel (contraction–expansion arrays) and fluid dynamic forces, including lift, drag, centrifugal, and Coriolis forces. Due to the rotation of the proposed platform, centrifugal and Coriolis forces act as driving forces for the fluid flow and the resultant fluid dynamic forces for the lateral migration of particles are directly dependent on the angular velocity of the platform. To determine the optimal angular velocity in the separation unit, six different angular velocities of 500, 1000, 1500, 2000, 2500, and 3000 rpm were investigated. Contraction–expansion inertial cell separation devices benefit from the formation of two counter-rotating vortices in the contraction regions region of the channels. These regions play a crucial role in the lateral migration of particles as mentioned in Equations (10) and (11). Figure 4A shows velocity distribution and vortex formation in a cross-section of a contraction region of our proposed model at 2000 rpm. In Figure 4B, a vertical line in the middle of the same cross-section is depicted, and velocity profiles in the radial direction for each angular velocity along the mentioned line were determined. As shown in Figure 4C, there is a parabolic velocity profile for each angular velocity. For the simulation of the separation unit, mesh independency analysis was performed and the magnitude of lateral velocity profile (square root of ($v^{2}+w^{2}$)) along the midline of the cross-section was determined for different computational grids, as shown in Figure 4D. We validated that the results were independent of the grid used, as no significant difference amongst grids of varying size were observed. Moreover, based on our formulations for inertial lift forces in Equations (13) and (14), the calculations of lift force for each particle were performed for particle tracing in cell separation section. Figure 4E,F shows the lift force magnitude distribution and its vectors for CTCs with size of 20 µm in a cross-section of contraction and expansion regions of the channel. There is a symmetric distribution of lift force in the contraction region because of the parabolic flow velocity profile. In the expansion section of the device, the region in line with the contracted segments is dominated by inertial lift compared to the expanded region in which there is little lift force.

**Figure 5:**
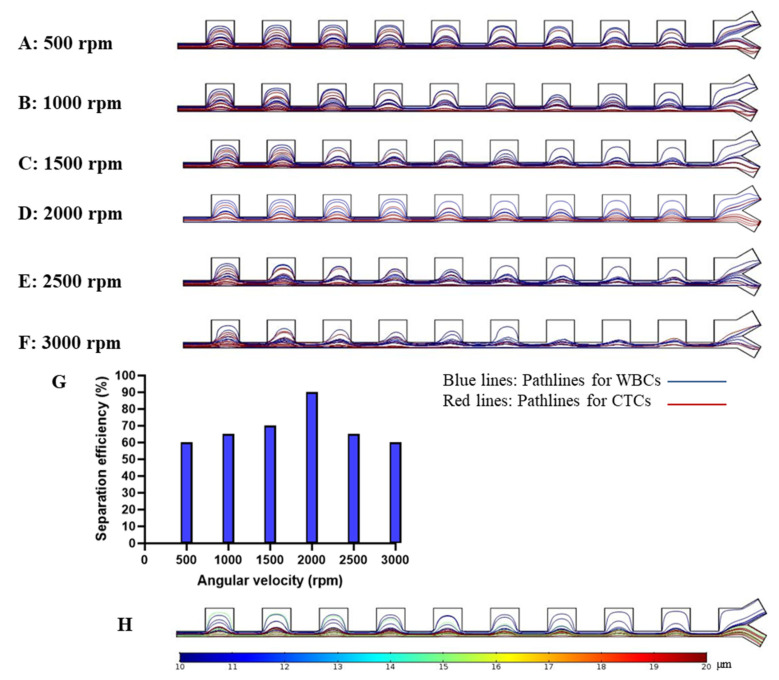
Particle tracking in proposed separation unit for different angular velocity. (**A**–**F**) Particles’ path lines and location at the outlets of the channel for different angular velocity. (**A**) 500 rpm, (**B**) 1000 rpm, (**C**) 1500 rpm, (**D**) 2000 rpm, (**E**) 2500 rpm, (**F**) 3000 rpm. Red lines show the path lines for CTCs and blue lines show the path lines for WBCs. (**G**) Separation efficiency for different angular velocity. (**H**) Path lines for CTCs with 15 and 20 µm diameters and WBCs with a 10 µm diameter.

After ensuring mesh independency and vortex formation in contraction regions, the governing equations for the particles’ motion were numerically solved. Different dominant forces, including centrifugal force, Coriolis force, inertial lift force, main flow drag, and dean drag forces were applied to the equation of motion for particles to determine their path through the channel. Though unrepresentative of the true ratio of CTCs to WBCs, the path of 40 total particles (20 WBCs and 20 CTCs) were simulated to validate performance based on size and to reduce computational cost. Both cell types were placed randomly in the inlet and were tracked while passing through the channel. Despite the skewed ratio of cell types, we still do not anticipate significant cell–cell interactions in the system due to the dilution of the cell suspension. The ratio was selected as a proof of concept purpose to illustrate the feasibility of size-based cell separation for two cell populations. Simulations were performed using different angular velocities and the resultant path lines for particles for each angular velocity are shown in Figure 5A–F. The proposed device was found to have high efficiency at the angular velocity of 2000 rpm, and 90% of the targeted cells (CTCs) in this angular velocity were directed to desired outlets.

Yield, or separation efficiency, is the most common parameter for evaluating the separation performance. It is defined as “the ratio of the number of targeted particles collected in the specific outlet to the total number of those particles in the inlet,” and this parameter was assessed for each angular velocity.

(20) $$\mathrm{Separation}\;\mathrm{efficiency}=\frac{\mathrm{Number}\;\mathrm{of}\;\mathrm{targeted}\;\mathrm{particles}\;\mathrm{collected}\;\mathrm{in}\;\mathrm{the}\;\mathrm{specific}\;\mathrm{outlet}}{\mathrm{Total}\;\mathrm{number}\;\mathrm{of}\;\mathrm{those}\;\mathrm{particles}\;\mathrm{in}\;\mathrm{the}\;\mathrm{inlet}}\times 100$$

By tracking each cell type related path and counting the particles collected in either outlet for CTCs, the separation efficiency was determined using Equation (20). Figure 5G shows the comparison of the separation efficiency at different angular velocities, and it shows that this device has the optimal separation efficiency at 2000 rpm. Particles in this angular velocity reach equilibrium positions at the outlets in a focused pattern in comparison to other angular velocities.

In order to consider CTCs with an average size of 15 µm, other simulations were performed to show the ability of our proposed cell separation platform to isolate the CTCs with the size of 15 and 20 µm from WBCs for the optimum angular velocity of 2000 rpm. As shown in Figure 5H the CTCs with two different sizes collected in the desired outlet and WBCs were directed into the upper outlet. Blue lines represent the path lines for WBCs, whereas the green and red lines show the path lines for CTCs with sizes of 15 µm and 20 µm, respectively. Separation efficiency for this condition was approximately 90%.

The proposed model took advantage of a contraction–expansion array for separation. This geometry forms vortices in the cross-section of the channel that lead to Dean drag forces to further induce lateral migration of particles already impacted by inertial lift forces. According to the results, operation at 2000 rpm led to the highest separation efficiency. For the separation unit with different angular velocities, the simulations showed that, at an angular velocity of 500 rpm, the centrifugal force and Coriolis forces are too low to result in dominant inertial lift and Dean drag forces to yield the lateral migration of particles. Additionally, it was observed that the angular velocity of 3000 rpm was too high as all particles were heavily deflected and shared a similar path leading to a low separation efficiency. The optimum efficiency in the separation unit was obtained at a speed of 2000 rpm.

In summary, our proposed cell separation unit successfully divides two types of particles, corresponding to WBCs and CTCs. The difference between the diameters of these cells, led to differential inertial lift and drag forces and, ultimately, different equilibrium positions to separate them. An optimal angular velocity of 2000 rpm was found to maximize the efficiency of this platform.

**Figure 6:**
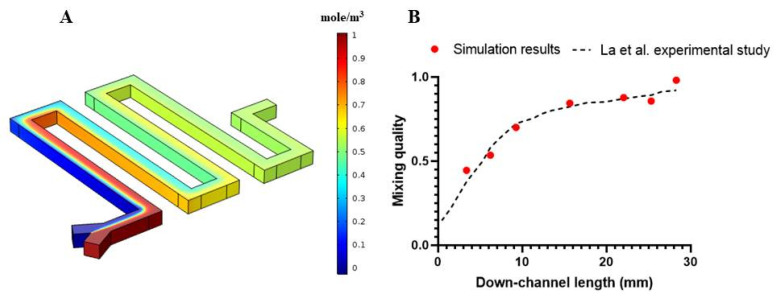
Validation of the mixing unit for our simulation method vs. La et al.’s experiment. (**A**) Concentration contour for the simulation model. (**B**) Comparison of mixing quality at different down-channel locations between numerical and experimental model.

After analyzing the performance of the proposed cell separation unit, we studied micromixer performance under different conditions. We developed two geometries, one a simple serpentine, and the other a serpentine with obstacles. To validate our numerical approach for determining concentration distribution in the mixer unit, our numerical simulation approach was utilized to simulate the performance of the proposed micromixer in the experimental work by La et al. [60] using a 209.4 rad/s angular velocity. By applying the appropriate boundary conditions, the governing equations for fluid flow and convection–diffusion equations for concentration distribution were numerically solved in their respective geometry, as shown in Figure 6A. After ensuring the mesh independency by performing several simulations with a variable number of elements, Figure 6A shows the concentration contour for the entire geometry proposed by La et al. As shown in Figure 6B, there is good agreement between the calculated mixing index versus down-channel length in simulation results and experimental results that ensures our simulation validity.

**Figure 7:**
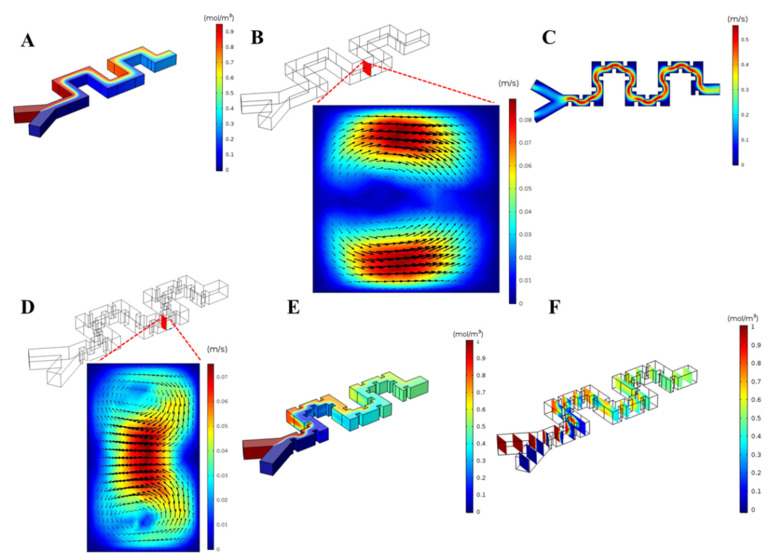
Mixer unit simulation. (**A**) Mixing pattern for serpentine micromixer without obstacles. (**B**) Vortex formation in serpentine channel without obstacles. (**C**) Velocity distribution for micromixer with obstacles at 2000 rpm. (**D**) Vortex formation in a serpentine channel with obstacles and related lateral velocity distribution within the cross-section. (**E**) Mixing pattern in proposed micromixer with obstacles at 2000 rpm. (**F**) Mixing pattern in the proposed micromixer in different cross-sections along the channel.

Due to the separation unit’s optimal performance at an angular velocity of 2000 rpm, we used the same conditions for the mixer unit first. After applying the boundary conditions, as mentioned in the above sections, the numerical simulation showed that the proposed serpentine micromixer without obstacles in the defined length (~2.5 mm) did not lead to high mixing quality, as shown in Figure 7A. The vortex formation in the micromixer without obstacles is shown in Figure 7B. To improve this efficiency, 40μm×40μm square-shaped obstacles, as shown in Figure 1D, were introduced to increase the contact area of the two fluids. We assume that the cell suspension has been diluted and that the alternations in fluid dynamics resulting from the passage of a CTC will be transient and negligible. Moreover, by adding the obstacles in the micromixer design to form secondary flows, the buffer reagents interact with cells sufficiently to lyse the cell and disperse the contents into solution. In the simulation including obstacles and an angular velocity of 2000 rpm, the flow was disturbed, and the mixing of fluids was enhanced by creating secondary flows around obstacles, as shown in Figure 7C,D. Notably, the pattern of the vortices varies significantly from the system without obstacles as two counter-rotating vortices are formed in the obstructed channel. As a result, the contact length of two fluids is increased and the formation of such vortices occur in more regions that leads to more diffusion of the two fluids within each other, compared to the micromixer without obstacles. As shown in Figure 7E, the solutions are nearly completely mixed at the outlet. Moreover, Figure 7F shows the concentration distribution in different cross-sections along the channel. By observing sequential planes in the radial direction along the channel, the concentration distribution leads to more uniform distribution and more mixing quality.

**Figure 8:**
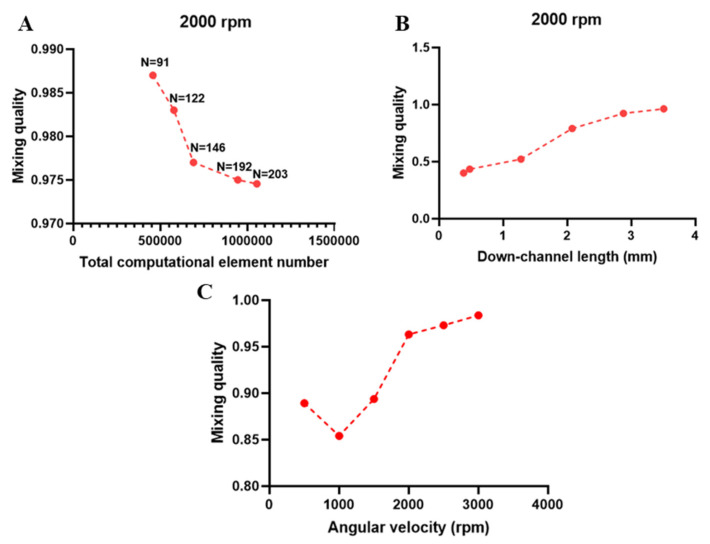
(**A**) Mesh independency analysis for mixer unit. (**B**) Mixing quality assessment along the down-channel length. (**C**) Mixing quality at different angular velocities.

For the mixer unit, mesh independency analysis was performed based on the mixing index at the outlet of the mixer, as shown in Figure 8A. A total number of 945,034 elements were used as the mixing index did not change significantly with finer sampling and smaller element sizes. The number of points considered in calculations of mixing quality (Equation (18)) in the outlet cross-section is presented in Figure 8B. Beyond N = 192, the mixing quality did not change significantly and the mesh was used as a benchmark for all other simulations. The mixing quality of the proposed micromixer with obstacles was calculated at different locations in the channel, as shown in Figure 8B. The mixing yield increased along the channel and, at the outlet, it reached approximately 98%. Moreover, mixing efficiency was investigated at the outlet of micromixers operated at different angular velocities, as shown in Figure 8C. Based on the simulated results, the angular velocity of 1000 rpm led to the lowest mixing quality, while augmented operation velocities yielded better mixing quality. The reason for this phenomenon is that, at low angular velocities, the Reynolds number is small, and the consequent secondary flows are rather weak. Within these regions, diffusion is the dominant mixing mechanism. Upon increasing angular velocity beyond a specific value, here 1000 rpm, which we name “threshold angular velocity”, the secondary flow generated due to the channel design and the imposed Coriolis force, becomes the dominant mixing mechanism. Therefore, in angular velocities more than this threshold, the *M.Q*. is continuously enhanced. Since our proposed separation unit had optimal efficiency at 2000 rpm, mixer unit simulations, also demonstrating high (~98%) mixing quality at the same angular velocity, show that operation at 2000 rpm would be viable for both processes, as shown in Figure 8C.

## 4. Limitations and Suggestions

In the present study we considered blood cells and CTCs as rigid, spherical particles. However, white blood cells and CTCs are not perfect spheres and are deformable under stress. Moreover, to reduce the computational time and more easily study the size-based separation of these cells, we used an equal proportion of WBCs and CTCs for simulation though the ratio of CTCs in blood is very low. Performing a simulation with a more representative number of particles/cells may be useful prior to fabrication and use. Additionally, this study was performed by neglecting particle–particle interactions, which may yield interesting results with higher cell densities. Furthermore, other models of particle lift forces relying upon direct numerical simulation may also be explored. Finally, with respect to micromixing, we did not consider the presence of the CTCs in the channels due to the expected transient stability of the cells in lysis buffer; however, additional numerical simulation involving morphological changes in the particle in the channel may provide additional insight to the true behavior.

## 5. Conclusions

In this study, we designed and computationally tested the performance of an integrated centrifugal force-based microfluidic device with two functions: cell separation and mixing. The numerical simulation of an efficient microfluidic platform for isolating CTCs from blood samples was proposed, and our numerical approaches were validated by the experimental data of Morijiri et al. [38] for cell separation and La et al. [60] for mixing. In our proposed device, WBC and CTC-simulating particles were separated using a size-based and label-free approach. An angular velocity of 2000 rpm was shown to yield higher separation efficiency in comparison to other angular velocities in the range of 500 to 3000 rpm. The output of this separation unit had a high efficiency (~90%). Moreover, in this platform, a micromixer was designed for CTC cell lysis and DNA extraction. We used a serpentine microchannel with and without embedded obstacles and investigated mixing performance in these micromixers. It was observed that adding obstacles increased the mixing quality significantly due to increasing the contact length of two fluids and the formation of secondary flows in different regions along the channel. In addition, the mixing quality of our proposed micromixer was assessed at different angular velocities, and 2000 rpm, which showed the maximum efficiency for separation unit, results in efficient performance (98% mixing quality) of the mixing unit as well. Therefore, our proposed integrated platform is shown to operate well at 2000 rpm for both cell separation and micromixing. Moreover, the fabrication of this integrated platform can be used in practical diagnostic applications in resource-poor settings.

## Figures and Tables

**Table 1:** Correction coefficients for inertial lift force proposed by Liu et al. [56].

AR	C_1_	C_2_
1	0.056	0.03
2	0.021	0.018
4	0.023	0.127
6	0.068	0.135
